# Surface chemical changes analysis of UV-light irradiated Moso bamboo (*Phyllostachys pubescens* Mazel)

**DOI:** 10.1098/rsos.180110

**Published:** 2018-06-20

**Authors:** Hai-xia Yu, Xin Pan, Man-ping Xu, Wei-ming Yang, Jin Wang, Xiao-wei Zhuang

**Affiliations:** 1 Zhejiang Academy of Forestry (Zhejiang Provincial Key Laboratory of Biological and Chemical Utilization of Forest Resources), 399# Liuhe Road, Xihu District, Hangzhou, Zhejiang 310023, People's Republic of China; 2 Zhejiang Forestry Product Testing Station, 399# Liuhe Road, Xihu District, Hangzhou, Zhejiang 310023, People's Republic of China

**Keywords:** bamboo, UV irradiation, lignin content, photodegradation, discolour

## Abstract

Photodegradation is one of the key factors that affect bamboo material application in the exterior environment. Photo radiation will cause chemical degradation, discoloration and cracks on the bamboo surface, thus resulting in weakened strength. The study imitated the accelerated weathering effect of Moso bamboo in sunlight by using UV 313 light. Results showed that after UV irradiation, lignin content decreased sharply. Lignin degradation products are commonly rich in double bonds conjugated with benzene rings; they absorb UV light and shift surface spectral absorbency from the visible to the UV region and play an important role in the first stage of reddish-yellow discoloration. The photochemical reactions were very rapid at the beginning and then slowed down after one week. The degraded products covered the surface and protected the inner layer from further degradation. The surface colour turned grey and lighter with erosion of degradation products when the experimental time was extended.

## 1. Introduction

Bamboo material, with high mechanical performance and fast growing properties (3–5 years to harvest), has been widely used for exterior applications in China such as outdoor flooring, benches, tables, wall decoration, pavilions, etc. [1]. However, during their utilization, bamboo materials are susceptible to solar radiation, which can initiate surface discoloration and cracking [2]. This is induced by surface chemical degradation [3]. When the degraded products from the cell wall material are washed away, the ageing process will progress to the deeper layers and leave a cellulose-rich greyish surface. This will reduce the commercial value and affect outdoor applications. With the rapid expansion of outdoor bamboo material, light ageing becomes one of the key factors that constrain bamboo applications. The mechanism of light ageing and the anti-light ageing technique have been of great concern to the bamboo industry.

There have been many studies on photoageing of wood, the main reasons for this being the degradation and transformation of lignin and extractives [3–5]. However, bamboo is a kind of highly lignified grass. Its lignin type, lignin content and extractives are quite different from those of wood. Bamboo is rich in phenol acids [6] and relatively high in lignin content (about 20–25%), and its lignin composition is typically H-G-S [6]. The difference in chemical composition will directly affect its light absorption characteristics. However, there have been few studies so far on the chemical changes in bamboo during UV irradiation.

To better reveal the photodegradation character of bamboo material, the present study exposed Moso bamboo to a UV 313 light source to imitate the accelerated effect of UV light in sunlight. The surface colour, lignin content and chemical composition were analysed. It was hoped that the study could further reveal the mechanism of discoloration due to the photo radiation of bamboo materials during their outdoor application.

## 2. Material and methods

### 2.1. Sample preparation

Four-year-old Moso bamboo (*Phyllostachys pubescens* Mazel) culms were cut from Zhejiang forestry academy; the culms were divided into eight strips and then the outside and inside layers were planed off (about 2 mm thickness) to get a smooth surface. The fresh bamboo strips were sliced along the outside surface into 75 µm thickness with a microtome. Bamboo strips and bamboo slices were fixed in a device, and then were covered by quartz glass and passed through 98% ultraviolet light and oven-dried at 35°C for 8 h. Then they were exposed to a 0.68 W m^−2^ (at 310 nm) UV light source under a relative humidity of 50% and temperature of 35°C for 56 days.

### 2.2. Colour measurement

Surface colour was monitored by a colorimeter (Data-color, CM-3600d, Japan) at 360–740 nm with a measuring head 8 mm in diameter (CIE Illuminant D 65), with an observation angle of 10°. The equipment was calibrated with standards provided by the supplier. The reflectance spectrum was converted to the K–M function as defined by expression [7].

$$F(\gamma_{\lambda })=\frac{K}{S}-\frac{{\left(1-\gamma_{\lambda }\right)}^{2}}{2\gamma_{\lambda }}$$

and

$$\gamma_{\lambda }=\frac{\gamma_{\left(\mathrm{sample}\right)}}{\gamma_{\left(\mathrm{standard}\right)}}$$

Here, *K* and *S* are the absorption and scattering coefficients, respectively, and *r* is the ratio of sample reflectance to the reflectance of a Whatman cellulose paper (no. 42) with a defined porosity [8]. Here, *γ*_(sample)_ is the sample reflectance and *γ*_(standard)_ is the reflectance of the cellulose paper (Whatman paper no 42.).

Colour changes are also analysed using the CIE Lab system, which is characterized by three parameters (L*, a* and b*), where L* represents the level of brightness (L* = 100 for pure white, L* = 0 for total black), a* represented red and green (+*a* for red, −*a* for green), and b* represented yellow and blue (+*b* for yellow, −*b* for blue). The total colour change (DE) was computed from the D_L_, D_a_ and D_b_ values using the following equation:

$$\Delta E^{*}=\sqrt{{\left(\Delta L^{*}\right)}^{2}+{\left(\Delta a^{*}\right)}^{2}+{\left(\Delta b^{*}\right)}^{2}}.$$

### 2.3. X-ray photoelectron spectroscopy analysis

The bamboo surface was directly exposed to a Kratos Axis Ultra spectrometer (PHI-5400, USA), using a monochromatic Al Ka X-ray source (*h*= 1486.6 eV) with a power of 225 W (15 kV voltage, emission current 15 mA), 284.8 eV pollution carbon (internal standard), minimum energy resolution of 0.48 eV, Ag (3 d5/2) and a minimum XPS analysis area of 15 µm.

### 2.4. Fourier-transform infrared spectroscopy spectra

Powder was gently scraped from the top surface of the bamboo strips with a sharp blade after UV irradiation. It was weighed out to 20 mg (accurate to 0.1 mg) and mixed with 3 g of KBr powder (spectroscopic grade) to get a 1 : 150 mixing ratio; then this was vacuum dried for 2 h. From this dried mixture, 700 mg was ground in an agate mortar, and pressed into transparent pellets on a hydraulic press. Spectra were recorded with a Nicolet I S10 spectrometer (Nicolet, USA) against pure KBr, with 4 cm^–1^ resolution, 64 scan times and a (400–4000) cm^–1^ spectral range. Special attention was given to peaks from 800 to 1800 cm^–1^.

### 2.5. Lignin content

The bamboo slices were cut into 1 mm long pieces, and 5 mg was weighed out with an electronic balance (accurate to 0.1 mg) and then placed into a 25 ml Teflon tube. Five millilitres of 1 : 4 (vol/vol) acetyl bromine/acetic acid was added, the tube was capped and then sealed with sealing film. The tube was placed in a 50°C water bath and shocked for 3 h. In the meantime, 10 ml 2 mol l^–1^ NaOH and 3 ml 0.5 mol l^–1^ hydroxylamine hydrochloride were added to a 50 ml volumetric flask. After 3 h, the tube was removed and its contents poured out into the 50 ml volumetric flask. To terminate the brominating reaction, acetic acid was added and diluted to the scale. The absorption value was then measured at 280 nm. At the same time, a blank solution was prepared [9]. The standard curve was obtained from a simple linear regression of alkali lignin standard solutions (with concentrations of 0, 10, 25, 50 and 100 mg l^–1^) and the corresponding absorbent values.

### 2.6. Gas chromatography–mass spectrometry analysis

Bamboo slices were cut into small pieces before and after 28 days of UV irradiation and 2 g was weighed out and soaked in dichloromethane solution for 12 h at room temperature and then extracted in Soxhlet at 55°C for 24 h. The extraction solution was vacuum condensed to about 3 ml. Analyses were carried out in a GC-MS (Agilent 7890 A) system using a 0.25 µm RTX-5MS column (30 m × 0.25 mm × 0.25 µm). Helium was used as a carrier gas at a flow rate of 1 ml min^–1^. The injector was maintained at 270°C. The column temperature was programmed as follows: 50°C (1 min), 5°C min^−1^ to 240°C (1 min) and 10°C min^−1^ to 280°C (10 min). A splitless injection mode was used with a split ratio of 30 : 1 and introduced 1 ml of sample. MS (full scan mode) was conducted as the following parameters: EM voltage of 1306 V, MS source at 230°C and MS quad at 150°C. The individual peaks were also compared with the Wiley computer mass library and calculated by peak area. The normalization method was used to calculate the relative percentage of each peak area, and the chemical composition was identified by the NIST library.

## 3. Results and discussion

### 3.1. Surface discoloration

**Figure 1.**
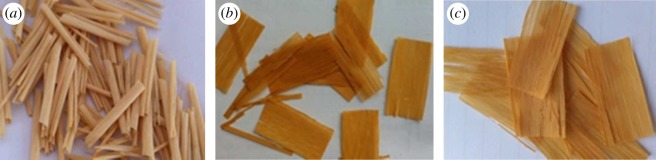
Moso bamboo slices before (*a*) and after 28 days (*b*) and 56 days of UV irradiation (*c*).

Figure 1 shows the visual observation of bamboo surface colour. It turned reddish-yellow after 28 days of UV irradiation, and then became light coloured after 56 days.

**Figure 2.**
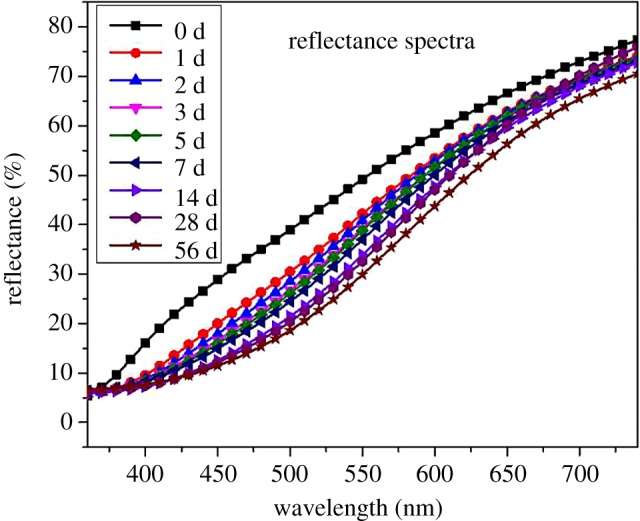
Reflection spectra of Moso bamboo.

**Figure 3.**
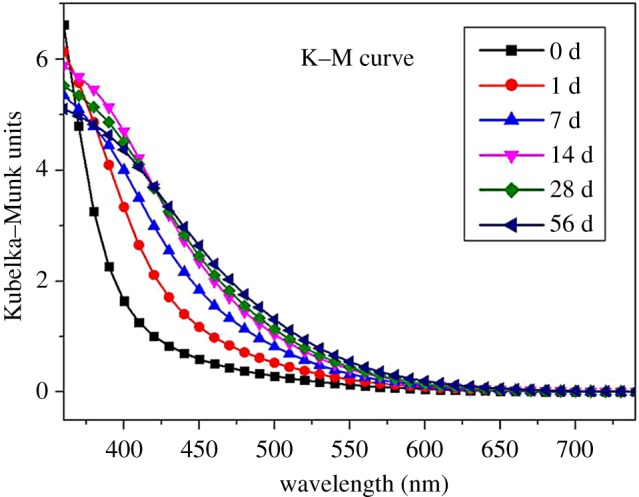
Evolution of K–M difference spectra for Moso bamboo as a function of light irradiation time.

Figures 2 and 3 show the reflectance spectra and the K–M curve after UV irradiation. In the range of 400–600 nm, the curve is raised. The rise in the K–M curve in the range of 400–415 nm indicates the formation of quinine structure products [10]. The K–M curve in the range of 360–600 nm may be associated with biphenyl, conjugated and C = O structures [11,12]. In this experiment, we used a colorimeter in visible light and failed to calculate the peak of the K–M curve. However, it is evident that the peaks are shifted to the UV region.

**Figure 4.**
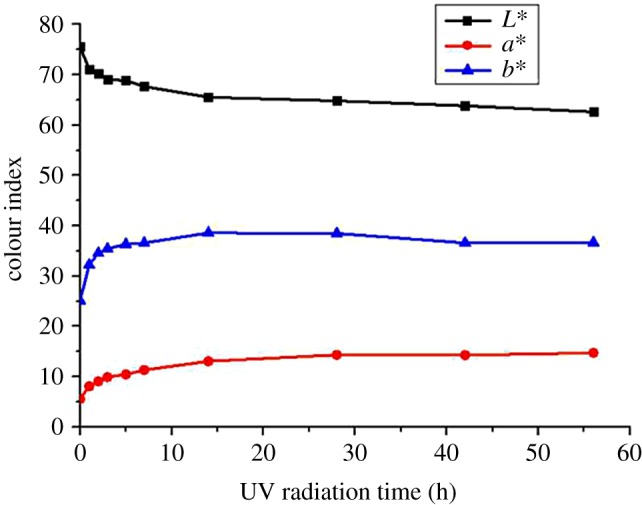
Colour parameters of Moso bamboo surface after UV irradiation.

Figure 4 shows that after photoageing treatment, the surface brightness L* of the bamboo was decreased, which made the colour darker; whereas a* and b* increased simultaneously, indicating a reddish-yellow development and showing more colour saturation.

It also can be seen that the colour parameters changed rapidly during the initial 5 days, then gradually decreased and tended to become stable after 14 days. Rapid changes in the early stage indicated a quick surface photochemical reaction. The reaction was initiated by a free radical reaction [13]. Free radicals need to diffuse from the surface to the inside. However, the degree of freedom of the internal molecular motion in solid bamboo was very small, so the reaction speed decreased; in addition, the deposition of degraded products would further form a protective layer.

### 3.2. X-ray photoelectron spectroscopy on elements of C and O

**Figure 5.**
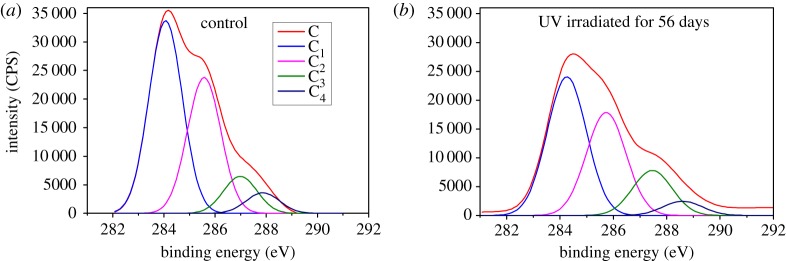
C_1s_ peaks of bamboo before (*a*) and after (*b*) 56 days of UV irradiation.

Surface elements were analysed by XPS. Figure 5 shows that C_1s_ was composed of four peaks. The binding energy of C_1_ was 284.06 eV; C_2_ was 285.57 eV; C_3_ was 286.98 eV; and C_4_ was 287.86 eV. C_1_ was mainly derived from lignin and wood extractives, while C_2_ mainly from cellulose.

Figure 5 shows that after UV light irradiation, the composition of carbon at the Moso bamboo surface changed. The C_1_ peak decreased, while the C_2_ and C_3_ peaks increased. This indicated that the carbon-binding energy increased, and became more stable.

**Figure 6.**
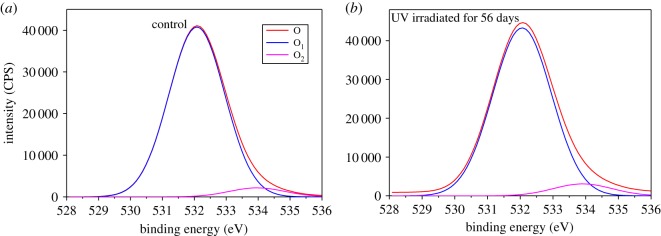
O_1s_ peaks of bamboo before (*a*) and after 56 days of UV irradiation (*b*).

Figure 6 shows that the O_1s_ peak was composed of two peaks. The binding energy of O_1_ was 532.20 eV, and that of O_2_ was 534.35 eV. After UV light irradiation, the O_1_ peak decreased and O_2_ increased.

**Figure 7.**
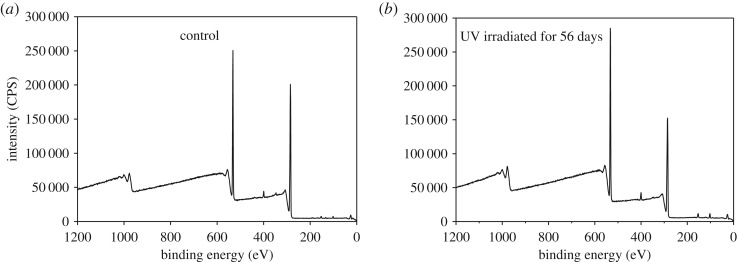
Wide scan XPS spectra of bamboo before (*a*) and after (*b*) 56 days of UV irradiation.

**Figure 8.**
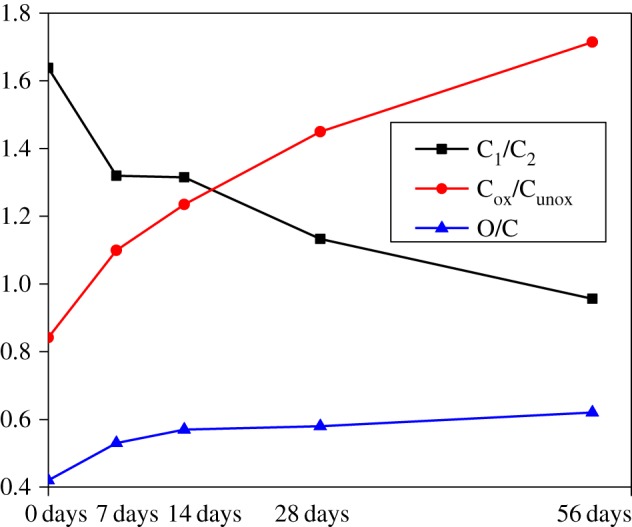
C(_1s_) and O/C proportion of bamboo after light irradiation.

Figure 7 shows the XPS wide scan map before and after UV irradiation. The peak was calculated to get the O/C ratio (figure 8). Figure 8 shows that the lower binding energy of C_1_/C_2_ declined and that of O/C increased as UV irradiation progressed. This indicates that oxidation occurred. According to Stark & Matuana [14], oxygenated and non-oxygenated carbon composition can be analysed. C_ox_/C_unox_ = C_oxygenated_/C_unoxygenated_ = (C_2_ + C_3_ + C_4_)/C_1_.

Table 1 shows that the relative content of C_1_ decreased continuously, especially in the first week; C_2_, C_3_ and C_4_ increased gradually as UV irradiation extended.

**Table 1.** Relative content of C and O in Moso bamboo before and after UV irradiation.

			relative content (radiation days)				
elements	binding type	binding energy/eV	0 days (%)	7 days (%)	14 days (%)	28 days (%)	56 days (%)
C_1_	C–C or C–H	284.06	54.30	47.63	44.75	42.46	36.84
C_2_	C–O	285.57	33.16	34.03	36.10	37.47	38.52
C_3_	O–C–O or C=O	286.98	9.85	10.51	11.96	13.34	14.78
C_4_	O–C=O	287.86	2.69	5.76	9.26	9.86	10.73
O_1_	O–C=O	532.2	94.65	96.63	94.57	92.65	91.90
O_2_	C–O–, C=O, C–O–C, O–C=O	534.35	5.35	3.37	5.43	7.35	8.10

### 3.3. Functional group changes due to UV irradiation

**Figure 9.**
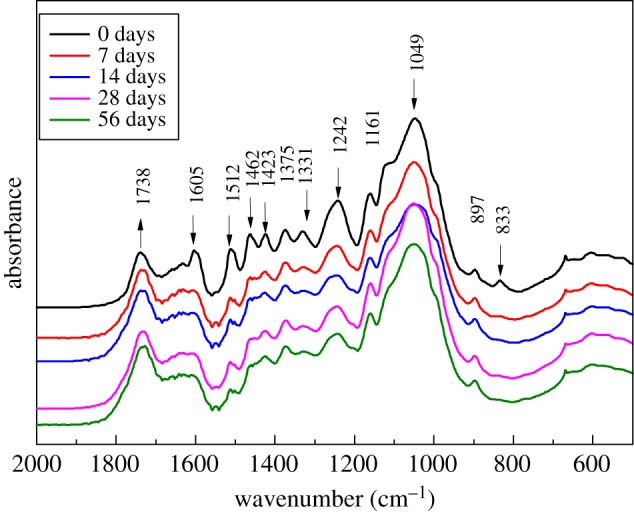
FTIR absorption spectra changes of Moso bamboo that occurred by artificial weathering.

The FITR spectrum in figure 9 shows that there are 12 main absorption peaks at the 600–1800 cm^–1^ wavenumber before light irradiation. After light irradiation, the peaks that related to lignin aromatic absorption all dropped significantly, among which the 1605, 1462, 1331 and 833 cm^–1^ peaks almost died out. By contrast, the peak at 1738 cm^–1^, which represented unconjugated carbonyl C=O stretching vibration of acetyl groups, became enhanced during the process. It was a sign of cleavage of acetyl groups and formation of new unconjugated carbonyl [15–17]. Peaks at 1375, 1161 and 897 cm^–1^, representing the absorption of polysaccharides such as cellulose and hemicelluloses, remained nearly unchanged during light irradiation. The loss of the peak intensity was also clear for 7 days of light exposure which meant that photochemical changes were quick at the beginning. The functional group changes then slowed down; this is probably because the degraded products covering the surface protected the inner layers from further degradation.

The peak at 833 cm^–1^, which represented G-type lignin, almost disappeared at 7 days, but the peaks of 1331 and 1242 cm^–1^, which represented S-type lignin, remained almost unchanged even after 56 days. It can be predicted that G-type lignin was more sensitive to UV light than S-type lignin.

### 3.4. Lignin degradation

**Figure 10.**
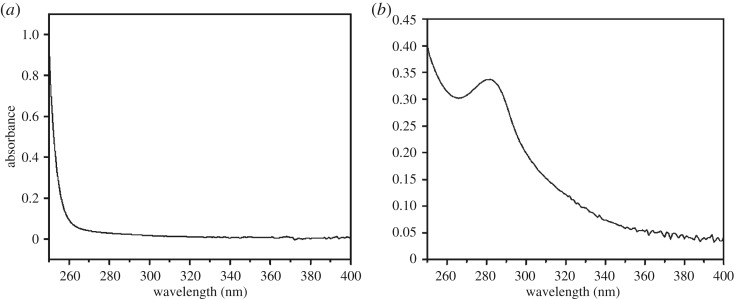
UV absorbance spectral characters of bamboo lignin ((*a*) blank and (*b*) bamboo lignin).

**Figure 11.**
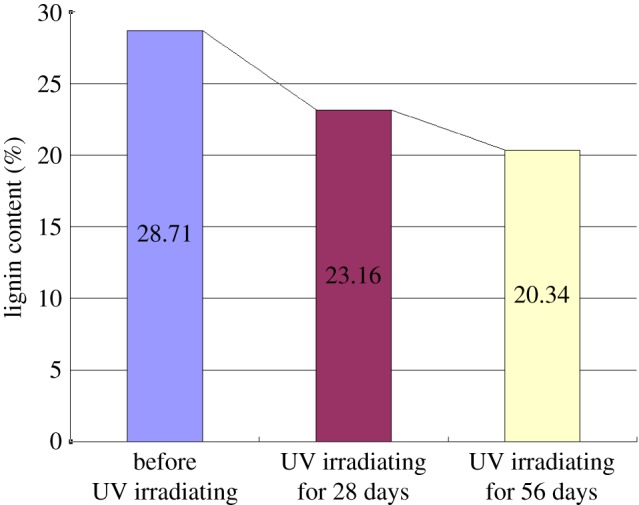
Lignin content of bamboo before and after UV irradiation.

Lignin content was accurately measured by the acetyl bromide method. The UV absorption spectrum is shown in figure 10. The acetyl bromide method proved to be an ideal method to evaluate trace lignin content changes during UV irradiation. Figure 11 shows that the lignin content of bamboo decreased after UV irradiation. The decrease rate was much quicker during days 0 to 28 than during days 28 to 56, and lignin degradation played an important role in the first stage of surface reddish-yellow discoloration.

### 3.5. Gas chromatography–mass spectrometry analysis

**Figure 12.**
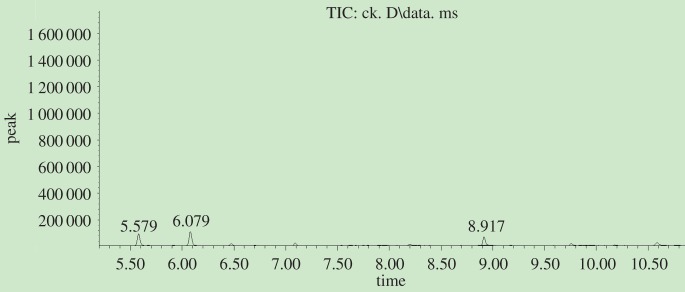
GC-MS chromatogram of methylene dichloride extraction from Moso bamboo before light irradiation.

**Figure 13.**
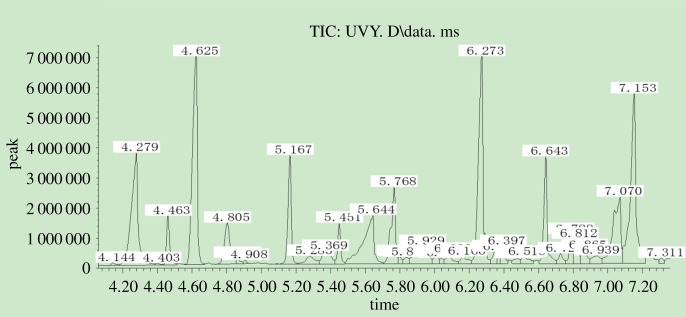
GC-MS chromatogram of methylene dichloride extraction from Moso bamboo after UV irradiation.

The decrease in lignin content was induced by photodegradation during UV irradiation. Figure 12 shows there were some lignin monomers and aromatic units in untreated bamboo (table 2), but the relative contents were very low. However, figure 13 identified more and relatively high contents of extractives. Most of the extractives were generated from lignin photodegradation.

**Table 2.** Main composition of methylene dichloride extraction from Moso bamboo before UV irradiation.

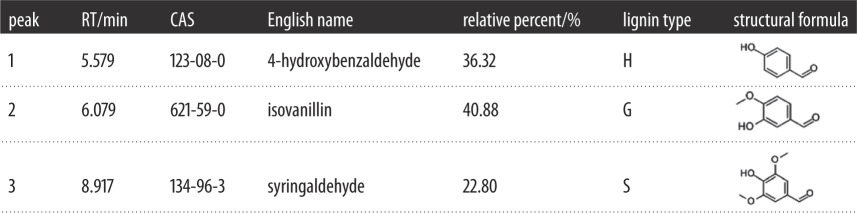

From table 3 we can see that the degraded products can be classified into six categories: aldehydes, ketones, esters, phenols, acids and biphenyls. The relative percent of *p*-hydroxybenzaldehyde, isovanillin, 4′-hydroxyacetophenone, 4′-hydroxy-3′-methoxyacetophenone, 3-hydroxy-4-methoxybenzoic acid, 4-*tert*-butylcatechol, 3,5-dimethoxybenzoic acid, syringaldehyde, acetosyringone, syringic acid and β-hydroxypropiovanillone were much higher for UV-irradiated bamboo. The degradation products were mainly from the breakdown of lignin macromolecules, and they were rich in conjugated double bonds with benzene rings. Conjugated double bonds can absorb UV light and change the surface spectral absorbent character, which shifted the surface colour from the visible to the UV region and played an important role in surface discoloration. The photodegraded products then covered the surface and prevented the layer beneath from undergoing degradation. However, those products were easily eroded away by wind and water [18], and subsequently, lignin degradation would progress into the deeper layers, and thus the surface changed from a dark to light colour.

**Table 3.** Main composition of methylene dichloride extraction from Moso bamboo after UV irradiation.

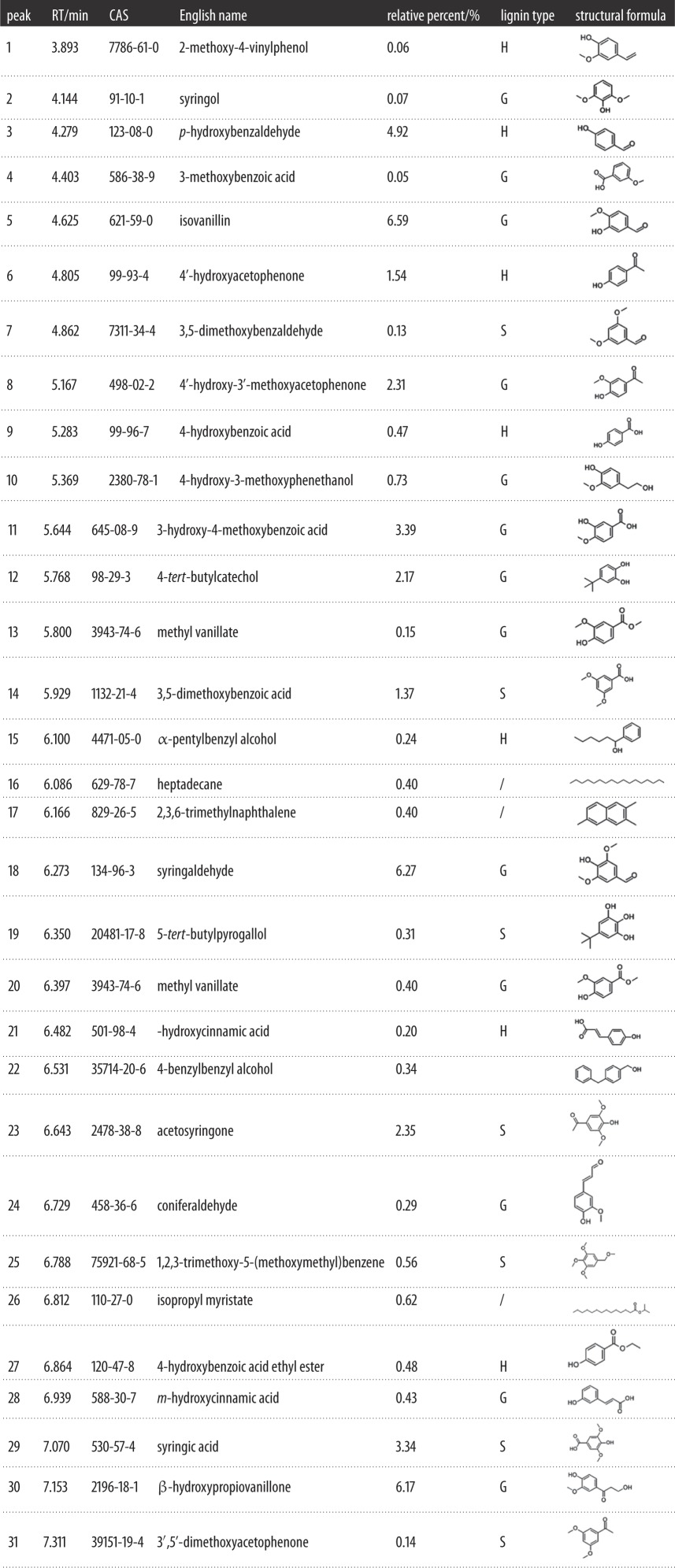

According to the relative proportion of the three units S, G and H in table 1, the percentage of each structural unit was H: 7.9%, G: 29.02%, S: 8.20% and the ratio of G : S was 3.67 : 1.04; it was much higher than before treatment (G : S = 9.51 : 8.08). It can be predicated that G-type lignin was more vulnerable to photodegradation than S-type lignin, and this was in agreement with the FTIR analysis in this paper as well as in previous studies on wood [10,18,19].

## 4. Conclusion

After UV irradiation, a series of photochemical reactions took place on the top surface of Moso bamboo. During the process, lignin degraded, lignin content decreased and extractives increased, and the surface colour changes were closely related to the chemical composition. Colour parameters, lignin content, and XPS and FTIR changes all showed that the photochemical reactions were very rapid at the beginning and then slowed down after one week. FTIR and GC-MS analysis showed that G-type lignin was more sensitive to UV light than S-type lignin. The binding energy C increased and oxidation occurred, and C became more stable after UV irradiation.

## Data accessibility

The datasets supporting this article have been uploaded at https://figshare.com/account/home#/data (doi:10.6084/m9.figshare.6143234).
